# Critical review and synthesis of the epidemiologic evidence on formaldehyde exposure and risk of leukemia and other lymphohematopoietic malignancies

**DOI:** 10.1007/s10552-012-0055-2

**Published:** 2012-09-15

**Authors:** Harvey Checkoway, Paolo Boffetta, Diane J. Mundt, Kenneth A. Mundt

**Affiliations:** 1 Department of Environmental Health, School of Public Health and Medicine, University of Washington, Box 357234, Seattle, WA 98195 USA; 2 Mt. Sinai School of Medicine, Institute for Translational Epidemiology, New York, NY 10029 USA; 3 ENVIRON International Corporation, Boston, MA 02110 USA; 4 ENVIRON International Corporation, Amherst, MA 01002 USA

**Keywords:** Formaldehyde, Leukemia, Lymphoma, Lymphohematopoietic malignancies, Epidemiologic review, Causation

## Abstract

**Purpose:**

Recent epidemiologic studies indicate elevated risks for some lymphohematopoietic malignancies (LHM) related to formaldehyde exposure. We performed a systematic review of literature to assess the strength and consistency of associations.

**Methods:**

We summarized published literature in the PubMed database of the National Library of Medicine during 1966–2012. Literature was categorized according to study design and population: industrial cohort studies, professional cohort studies, and population-based case–control studies.

**Results:**

Findings from occupational cohort and population-based case–control studies were very inconsistent for LHM, including myeloid leukemia. Apart from some isolated exceptions, relative risks were close to the null, and there was little evidence for dose–response relations for any of the LHM.

**Conclusions:**

At present, there is no consistent or strong epidemiologic evidence that formaldehyde is causally related to any of the LHM. The absence of established toxicological mechanisms further weakens any arguments for causation. To be informative, future epidemiologic research should improve on formaldehyde exposure assessment and apply modern diagnostic schemes for specific LHM.

## Introduction

Formaldehyde (CH_2_O) is a simple one-carbon molecule, found in most human and other living cells as a normal product of the metabolism of serine, glycine, methionine, and choline, and is generated in the demethylation of N-, O-, and S-methyl compounds. It is also an essential intermediate in the biosynthesis of purines, thymidine, and various amino acids [1]. Consequently, formaldehyde is present in virtually all cells in the body at varying concentrations.

Formaldehyde is also produced commercially and is valuable as a biocide, preservative, and basic chemical in the manufacture of common materials such as plastics, building materials, glues and fabrics, and many household and consumer products, including medicines, health, and beauty aids. Formaldehyde is also a product of organic matter combustion.

Common exposure sources include some laboratories, indoor air (e.g., carpets), vehicle emissions, cigarette smoke, and workplaces manufacturing or using resins, various wood products (e.g., particle board), adhesives, textiles, and numerous other consumer products [2]. High concentrations of formaldehyde were found inside some of the temporary housing units built for victims of hurricane Katrina in the US in 2008, which raised the public awareness of the chemical and its potential acute health effects [3, 4].

Inhalation is the predominant route of exposure to exogenous formaldehyde. Following inhalation, formaldehyde rapidly reaches cells in the upper respiratory tract and reacts virtually instantaneously with primary and secondary amines, thiols, hydroxyls, and amides [5]. Formaldehyde is swiftly metabolized by erythrocytes [6–9]. Formaldehyde forms adducts with DNA and proteins and also produces DNA cross-links [10].

The most common acute health effects of exposure to formaldehyde include eye and upper respiratory tract irritation. Reversible declines in lung function have also been observed, although the evidence that it causes asthma and other chronic respiratory diseases is inconsistent [11]. There is inadequate evidence to assess other potential adverse effects of formaldehyde in humans, such as immunotoxicity, neurotoxicity, and reproductive and developmental toxicity [12, 13].

### Carcinogenicity of formaldehyde

Concerns about the carcinogenicity of formaldehyde were prompted in the early 1980s by the induction of nasal tumors in rats exposed at high concentrations [14–17]. As a consequence, the focus of early epidemiologic studies was on nasal cancer, based on the understanding that formaldehyde is rapidly metabolized at the site of contact (i.e., nasal passages and cavity) [18–20]. Consequently, associations between formaldehyde exposure and other malignancies in humans were reported, including nasopharyngeal carcinoma (NPC), lung cancer, lymphohematopoietic malignancies (LHM), mainly leukemias, and other cancers such as brain, colon, and prostate [21, 22]. Epidemiologic studies on formaldehyde exposure and LHM risk are reviewed in detail below.

In 2006, the International Agency for Research on Cancer (IARC) conducted a comprehensive review of the literature and classified formaldehyde as a known (i.e., Group 1) human carcinogen, based on sufficient evidence for NPC. The evidence for leukemia was considered suggestive [23]. In 2009, IARC conducted an abbreviated updated review of all Group 1 chemicals, including formaldehyde [24], in which the epidemiologic evidence for leukemia—specifically myeloid leukemias—was classified as sufficient. The US National Toxicology Program similarly classified formaldehyde as a known human carcinogen [25]. The US Environmental Protection Agency (EPA), in its draft Integrated Risk Information System (IRIS) report on formaldehyde, concluded that existing epidemiologic evidence supported a causal association with LHM as a group and specifically for myeloid leukemia [26]. A special committee of the US National Research Council of the National Academies critically reviewed the EPA draft IRIS report and found the causal conclusions for LHM to be inadequately supported [27].

We undertook a critical, systematic, and comprehensive review and synthesis of the epidemiologic literature on formaldehyde and risks of the LHM. Our review is more thorough than that produced by the National Research Council [27], which focused on literature summarized in the EPA draft IRIS document. Our objectives were to characterize the overall strength and consistency of the evidence to guide causal interpretations and to recommend research improvements that would extend knowledge on this important public health and scientific issue.

## Methods

Our methods were consistent with those used by IARC [28] and others [29–31]. Briefly, we identified published, peer-reviewed epidemiologic studies specifically addressing formaldehyde exposure and risk of the LHM. Searches were conducted in PubMed, the US National Library of Medicine’s primary research tool that indexes most of the world’s health and medical peer-reviewed journals since at least 1966. All years indexed were searched to identify these studies using the following key words in various combinations: cancer, leukemia, non-Hodgkin’s lymphoma, lymphoma, lymphocytic, Hodgkin’s lymphoma, hematopoietic, multiple myeloma, hematological neoplasm, formaldehyde, embalmer, garment, laboratory workers, epidemiology, case–control, cohort, case-referent, occupational, chemical, exposure, risk, review, meta-analysis, and commentary. We identified a total of 1,441 potentially relevant articles from the literature searches. Of these articles, 126 were retained as relevant to formaldehyde exposures and LHM. Articles were excluded if they (1) were not epidemiological studies, (2) did not focus on formaldehyde, (3) focused on outcomes other than cancer, or (4) did not present results for specifically for LHM. Additionally, references cited in other publications, including reviews, were checked to ensure the thoroughness of the literature review. We did not attempt to identify unpublished reports. The final review included a total of 37 articles—22 cohort studies and 17 case–control studies.

We comprehensively reviewed the identified literature, including studies of occupational groups and population-based case–control studies of specific LHM that presented results for formaldehyde-related exposures. Most emphasis was placed on findings from occupational cohort studies, which, because of the greater potential for exposure to substantial concentrations of formaldehyde, provide the best evidence for possible associations. We limited the review to the most recent updates of occupational studies, although we include findings from earlier reports where results have changed materially with successive updates.

Defining the outcome of interest is an important aspect of the design of epidemiologic studies, and the LHM are particularly challenging in this regard. Much of the information about LHM and formaldehyde exposure derives from mortality data in occupational cohort studies that spanned several LHM classification schemes. The principles of the nosological classification of this group of neoplasms have changed during the past 40 years, following the increasing understanding of the pathological and clinical characteristics of the different diseases. The most substantial changes in the International Classification of Diseases (ICD) have occurred for the non-Hodgkin lymphomas (NHL). Until the 9th Revision of the International Classification of Diseases (ICD), NHL was classified under two rubrics: “lymphosarcoma and reticulosarcoma” and “other neoplasms of the lymphoid tissue” (Hodgkin lymphoma had a separate code) [32]. In ICD-10, which follows a new WHO classification, chronic lymphocytic leukemia (CLL), the most common type of leukemia among the elderly, is classified as a form of NHL, and other changes were made to the classification of NHL. The InterLymph Consortium of lymphoma epidemiology has made an effort to adapt the last two versions of the WHO classification to epidemiologic studies, following a hierarchical approach [33, 34]. Unfortunately, the majority of epidemiologic studies, in particular occupational cohort studies, which based outcomes on death certificates, do not follow the WHO classifications (or its InterLymph adaptation).

We present and discuss findings for specific LHM to the extent allowed by published data. We do not discuss results for all LHM combined because diseases in this group are clinically and pathologically heterogeneous, and thus probably etiologically distinctive.

**Fig. 1:**
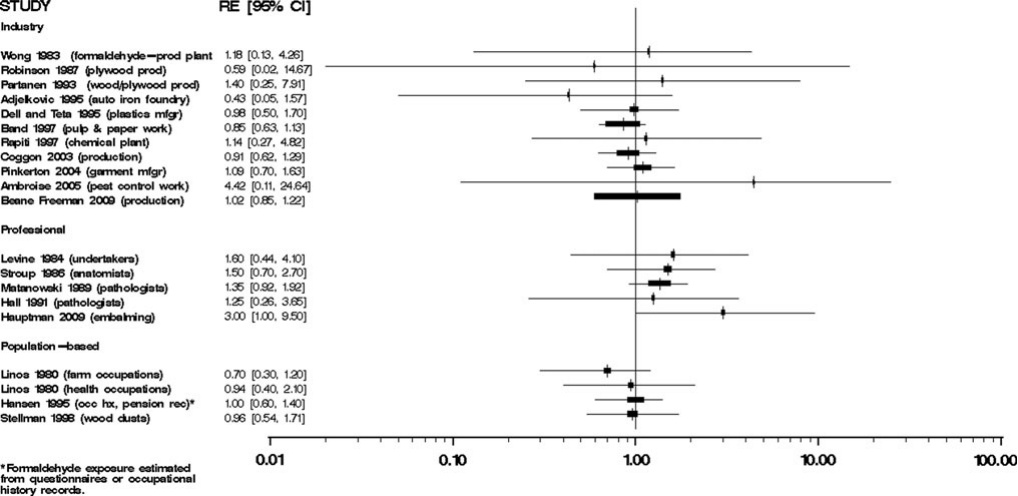
Forest plot of formaldehyde exposure and leukemias

**Fig. 2:**
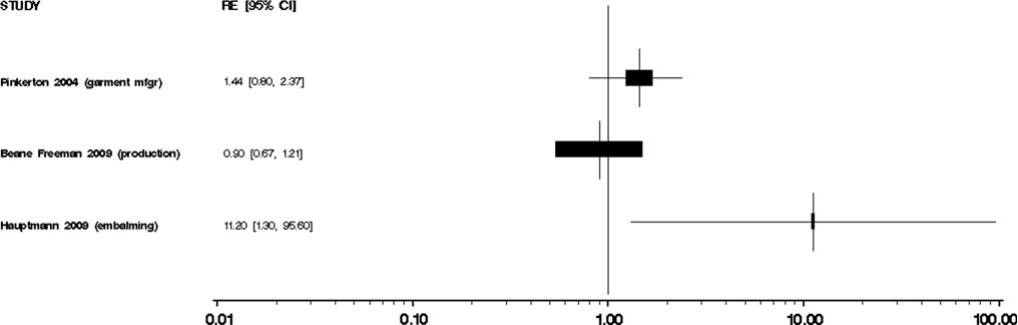
Forest plot of formaldehyde exposure and myeloid leukemia

**Fig. 3:**
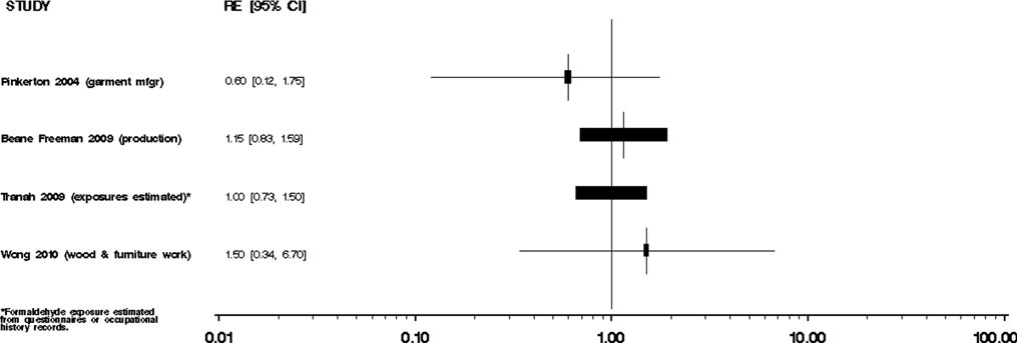
Forest plot of formaldehyde exposure and chronic lymphocytic leukemia

**Fig. 4:**
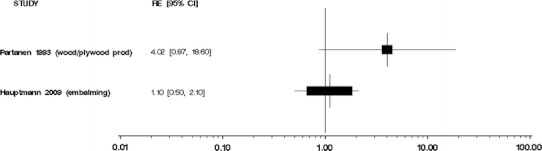
Forest plot of formaldehyde exposure and lymphomas

**Fig. 5:**
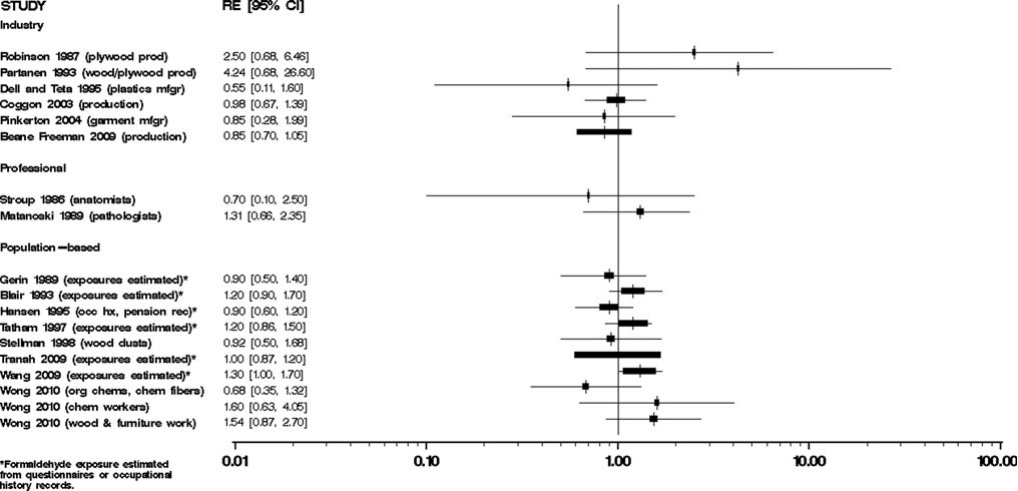
Forest plot of formaldehyde exposure and non-Hodgkin lymphoma

We did not perform meta-analyses because our evaluation of the individual studies determined that the literature is too heterogeneous, that is, inconsistent, with respect to disease classification and exposure assessment, and therefore, quantitative risks are not appropriately combined. Moreover, the number of independent studies with comparable exposure circumstances (i.e., the same industry or occupation) and similar exposure assessments was too small to justify meta-analyses of these subsets of results. We were especially concerned about combining studies of different groups of workers with poorly characterized circumstances of exposure to formaldehyde. Several previous meta-analyses [35–38] have been performed, yielding variable conclusions, which may result from different methods and the underlying heterogeneity of exposure and health outcome data specificity and validity among published studies. In our opinion, the apparent gain in precision from a meta-analysis would be offset by problems in the interpretation of the summary results. We do, however, provide Forest plots of overall study findings as Figs. 1, 2, 3, 4, and 5.

### Epidemiologic literature

Associations between formaldehyde exposure and the LHM have been investigated among anatomists, pathologists, embalmers, and industrial workers involved in the manufacture and use of formaldehyde and formaldehyde-containing products, such as resins, adhesives, wood products, fabrics, and garments. Formaldehyde has also been examined as a risk factor in numerous studies conducted in the general population, including population-based case–control studies and analyses correlating occupations with LHM incidence and mortality. Accordingly, we present summaries of literature in tabular form separately for the following categories: cohort studies of industrial workers, cohort studies of professional workers, and population-based cohort and case–control studies.

Among all available literature, we regard two large occupational cohort studies as most informative because of the cohort design, greatest likelihood of exposure, quantification of exposure, and minimized bias and confounding. These are mortality studies of (1) a cohort of employees of ten US factories that produced or used formaldehyde, conducted by the US National Cancer Institute (henceforth termed the “NCI producers study”) [39]^1^ and (2) a cohort of employees of six UK factories engaged in the production of resins, adhesives, and formalin (henceforth termed the “UK producers study”) [48].^2^

A second group of occupational studies that we regard as less informative includes a cohort of US garment workers [51–53]^3^ and a case–control analysis of deaths among US embalmers and funeral directors [54] that was based on a series of earlier proportionate mortality studies [21, 55, 56]. The study base in which the nested case–control study of LHM in the US embalmers and funeral directors study was conducted was poorly defined [54], and the formaldehyde exposure assessment in the garment workers study [53] was less specific and detailed than in the two “producers” cohort studies.

The remaining occupational studies reviewed were those conducted among cohorts of undertakers [57], pathologists [58], anatomists [59, 60], wood industry workers [61–63], and general chemical industry workers [20, 64–67]. In these studies, formaldehyde exposure was less certain than in aforementioned occupational cohort studies and, in many cases, was inferred from job title or work area.

The other major categories of epidemiologic studies reviewed were community-based cohort and case–control studies and general population surveys, which also provide limited information on formaldehyde exposure and LHM risks. Exposure assessment in these studies was generally based on crude exposure metrics, such as “low” versus “high” exposure probability combinations of heterogeneous job titles. Details of study design and exposure assessment for the studies reviewed are summarized in Table 1.

**Table 1:** Studies of formaldehyde and lymphohematopoeitic malignancies and exposure metrics

Study	Location	Occupational group	Cohort size	Follow-up	Relevant exposure metrics	Comments
Occupational cohort studies						
Fayerweather [64]	USA	8 Formaldehyde-producing or formaldehyde-using plants	481	1957–1979	Latency period (years, highest category: ≥20 years) Source of work history Pay class Duration of exposure (years, ≥5 years) Frequency of exposure Frequency and level of exposure Cumulative exposure index	Jobs were categorized into three exposure categories: continuous-direct, intermittent, and background. Exposure potentials were extrapolations based on air recent/past air monitoring, statements from long-term employees, knowledge of odor/sensory irritation thresholds, knowledge of past process changes, engineering/personal controls. Highest category of continuous exposure: Level 3; 8-hr TWA concentrations of ≥2.0 ppm Highest category of intermittent exposure: “High:” jobs permitting a worker to be exposed to peak formaldehyde concentrations ≥2.0 ppm Almost all exposure categories analyzed with respect to various latency period categories.
Wong [20]	USA	Formaldehyde-producing chemical plant	2,026	1940s–1977	Exposed/non-exposed Date of hire Latency period (years, highest category: 20 years) Length of employment (highest category: ≥20 years)	Analysis does not include detailed information re: individual work histories, exposures.
Levine [57]	Canada	Undertakers	1,477	1928–1977	Exposed/non-exposed	Exposure levels assayed from breathing zone of embalmers from seven US funeral homes, but not included in analysis. Expected deaths before 1950 determined by applying age- and calendar-year-specific mortality rates of men from the 1950–1977 cohort.
Liebling [86]	USA	Chemical plant	24	1976–1980	Exposed/non-exposed	Exposure to formaldehyde estimated from work histories.
Bertazzi [65]	Italy	Resins-manufacturing plant	1,332	1959–1980	Exposed/non-exposed Type of exposure (formaldehyde, other, unknown)	Jobs categorized into types of exposure (exposed to formaldehyde, exposed to other compounds, exposure unknown). Sampling data was not suitable for use in estimating exposures. Analysis for other cancers included analysis by year since first employment, length of employment, and years since first exposure.
Hagmar [87]	Sweden	Chemical plant	664	1942–1979	Exposed/non-exposed Duration of employment, induction/latency period (higher category: work for ≥6 months and an induction/latency period of ≥10 years) Previous chemical exposure (from history or records)	Exposures estimated from worker reports of time spent at various work processes involving possible exposure to the established or suspected carcinogens. Those working for ≥6 months and with an induction/latency period of ≥10 years placed into a restricted cohort.
Logue [88]	USA	Radiologists and pathologists	13,537	1962–1972	Exposed/non-exposed	Specific exposure measurements/assumptions not considered.
Stroup [86]	USA	Anatomists	2,317	1925–1979	Exposed/non-exposed	Exposures assumed based on duration of American Association of Anatomists membership and the time period in which anatomists joined the Association. Specific exposure measurements/assumptions not considered.
Robinson [61]	USA	Plywood mill workers	2,283	1945–1977	Exposed/non-exposed Years of employment Years of latency	Workers presumed to have formaldehyde exposures (based on job responsibilities) were placed into a separate subcohort.
Matanoski [60]	USA	Pathologists	6,411	1925–1978	Exposed/non-exposed	Specific exposure measurements/assumptions not considered.
Ott [66]	USA	3 chemical manufacturing facilities	129	1940–1978	Ever/never exposed (“ever”: employee worked for ≥1 day with a chemical in a specific work area) Work areas (+duration of work in these areas; highest category: ≥5 years) Chemical	Exposures assigned to work categories by using work histories, departmental and job assignment records, and historical information regarding process dates and descriptions. Individual contact with specific substances also estimated using employee work assignments and records with department usage for each substance. Further analysis conducted by chemical functional group
Hall [58]	UK	Pathologists	4,512	1974–1987	Exposed/non-exposed	Formaldehyde exposure assumed consistent among members of cohort due to the fact that cohort members had passed an examination for membership requiring some years of experience. No specific exposure measurements/assumptions.
Partanen [62]	Finland	Wood industry production workers	7,307	1945–1983	Type of exposure (yes/no to various categories of exposure)	Individual types of exposure (formaldehyde, wood dust, pesticides, chlorophenols, phenol, etc.) reconstructed from company records, interviews with personnel, questionnaires sent to next-of-kin.
Andjelkovich [89]	USA	Automotive iron foundry	3,929	1950–1989	Exposed/non-exposed	Occupational titles obtained from work histories categorized as high, medium, low, or no exposure to formaldehyde.
Dell [67]	USA	Plastics manufacturing, research, development facility	5,932	1946–1988	Exposed/non-exposed Salaried employees	Duration of employment used as an indirect measure of cumulative exposure. Specific exposure measurements/assumptions not considered. Additional analyses carried out for other cancers (by duration of employment, lag interval in years)
Rapiti [90]	Italy	Chemical plant	505	1954–1991	Ever/never work in a specific process	Specific exposure measurements/assumptions not considered
Stellman [63]	USA	Woodworkers, wood dust-exposed men	45,399	1982–1988	Type of employment Duration of wood dust exposure (years) Type of exposure (formaldehyde, asbestos, etc.)	Individual categorical exposures ascertained from completed checklists.
Coggon [48]	UK	6 Formaldehyde-producing or formaldehyde-using factories	14,014	1941–2000	Exposed/non-exposed “High” exposure (>2.0 ppm)	Exposures before 1970 estimated using later measurements and workers’ recall of irritant symptoms. Each job classified into one of five exposure categories (background, low, moderate, high, or unknown). Other exposure metrics (exposure category, years of employment, years since first employment in jobs with high exposures) included in analyses involving other outcomes. LHP-specific analyses, only included comparisons of exposed/non-exposed and high exposure/non-exposed populations.
Pinkerton [53]	USA	3 garment mfg. plants	11,039	1955–1998	Exposed/non-exposed Duration of exposure (years, highest category: ≥10 years) Time since first exposure (years, highest category: ≥20 years) Year of first exposure	Individual formaldehyde exposure levels determined for 549 (40 %) of then-current employees using a NIOSH sampling method. Historic exposures were not available, not estimated.
Ambroise [91]	France	Pest-control workers	181	1979–2000	Exposed/non-exposed	Exposures estimated from administrative records for job histories, interviews with former and present workers on workplaces, historical description of activities and relevant information on exposure and working conditions, and linked to a job matrix.
Beane Freeman [39]	USA	10 Formaldehyde-producing or formaldehyde-using factories	25,619	1966–2004	Exposed/non-exposed Peak exposure (ppm, highest category: ≥4.0 ppm) Average intensity (ppm, highest category: ≥1.0 ppm) Cumulative exposure (ppm-yr, highest category: ≥5.5 ppm-years)	Exposures estimated from individual work histories, expert assessments of job and department titles and tasks associated with jobs by using current and past measurement data. Exposures estimated for jobs, plants, and calendar-time.
Hauptmann [54]	USA	Embalmers and funeral directors	168 LHP deaths, 265 other deaths from 6,808 total deaths	1960–1986	Duration of exposure (years, highest category: >34 years) No. of embalmings (highest category: >3,068) Cumulative exposure (ppm-h, highest category: >9,253 years ppm-h) Average intensity (ppm, highest category: >1.9 ppm) Time-weighted average intensity (ppm, highest category: >0.18 ppm) Peak exposure (ppm, highest category: >9.3 ppm) Ever vs. never embalming	Individual job and year-specific exposures were determined by a model matching interview responses to the results of a previous exposure assessment.

Study	Location	LHP outcome	No. cases, controls	Exposure assessment	Comments
Population-based study					
Linos [92]	USA	Leukemia (Acute lymphocytic, acute myelocytic, chronic lymphocytic, chronic myelocytic)	138 cases, 176 controls	Analyses were specific to occupational group (farm- and health-related occupations); occupational information ascertained from medical records.	Case–control study
Boffetta [93]	USA	Multiple myeloma	128 cases, 512 controls	Exposures estimated from questionnaire responses.	Case–control study
Gerin [94]	Canada	Hodgkin’s lymphoma, Non-Hodgkin’s lymphoma	53 HL and 206 NHL cases, 533 general population controls, 2,599 cancer controls	Occupational exposures estimated from interviews. Based on review of interviews, experts assigned to each job indices of probability, frequency, and intensity of exposure to multiple agents.	Case–control study
Heineman [95]	Denmark	Multiple myeloma	1,098 cases, 4,169 controls	Occupational histories obtained from pension records and tax forms. Exposures estimated from occupational histories matched to a job-exposure matrix.	Case–control study
Pottern [96]	Denmark	Multiple myeloma	1,010 cases, 4,040 controls	Occupational histories obtained from pension records and tax forms. Based on the obtained information, subjects were categorized by likelihood of exposure to various substances including formaldehyde.	Case–control study
Blair [97]	USA	Non-Hodgkin’s lymphoma	622 cases, 1,245 controls	Exposures estimated from interviews linked to a job-exposure matrix.	Case–control study
Hansen [71]	Denmark	Non-Hodgkin’s lymphoma, Hodgkin’s lymphoma, leukemia	91,182	Occupational histories obtained from pension records. Those with occupational histories were stratified into “low” exposure or “above baseline” exposure groups based on job title and work at companies known to use/have used formaldehyde.	Cohort study
West [98]	UK	MDS	400 cases, 400 controls	Occupational and environmental exposures estimated from interviews and investigations of jobs associated with exposure. Subjects were categorized according to estimated intensities and sensitivities of exposure.	Case–control study
Band [99]	Canada	Non-Hodgkin’s lymphoma, multiple myeloma, Hodgkin’s disease, leukemia	30,157	Exposures were estimated using the time since subjects were first employed, duration of employment, and the type of mill worked in.	Cohort study
Tatham [100]	USA	Non-Hodgkin’s lymphoma	1,048 cases, 1,659 controls	Exposures estimated from interviews.	Case–control study
Blair [70]	USA	Leukemia (AML, CML, ALL), MDS, CLL	513 cases, 1,087 controls	Exposure probability and intensity estimates ascertained from interviews and linked to a job-exposure matrix.	Case–control study
Nisse [101]	France	MDS	204 cases, 204 controls	Occupational exposures to formaldehyde and other chemicals/compounds estimated from interviews.	Case–control study
Tranah [102]	USA	Non-Hodgkin’s lymphoma	1,591 cases, 2,515 controls	Exposures estimated from interviews linked to a job-exposure matrix.	Case–control study
Wang [72]	USA	Non-Hodgkin’s lymphoma	601 cases, 717 controls	Exposures estimated utilizing a questionnaire linked to a job-exposure matrix.	Case–control study
Wong [103]	China	NHL	649 cases, 1,298 controls	Samples obtained, exposures estimated from questionnaires. Exposure assessment conducted for benzene. Reported results specific to type of industry.	Case–control study

## Results

### Summary of leukemia findings

The findings for the occupational cohort studies with leukemia outcomes are summarized in Table 2. The two most influential studies are considered first. Based on comparisons with national rates, no excesses for all leukemia (standardized mortality ratio (SMR) 1.02, 95 % confidence interval (CI) 0.85–1.22) or myeloid leukemia (SMR 0.90, 95 % CI 0.67–1.21) were found in the most recent follow-up of the NCI producers’ study. Among the formaldehyde-exposed portion of the cohort, there was a weak trend of relative risk (RR) with peak exposure, for both all leukemias and myeloid leukemia, largely influenced by elevated RRs of 1.78 (95 % CI 0.87–3.64) for myeloid leukemias and 1.42 (0.92–2.18) for “other” (non-myeloid) leukemias in the highest peak exposure category. However, most of the trends and individual RR estimates were not remarkable or precise. The association for peak exposure and myeloid leukemia was considerably attenuated from the previous follow-up of the cohort, RR 2.79 (95 % CI 1.08–7.21, 14 cases, p-trend 0.02) at the highest peak category. Beane Freeman [39] corrected the results published in Hauptmann [47] that inadvertently omitted 1,006 deaths, including 22 LHM deaths. No clear associations with average or cumulative exposure were found in the corrected data for any of the leukemias. Null findings were reported for lymphatic leukemia and “other and unspecified leukemia” [39].

**Table 2:** Studies of formaldehyde exposure and leukemia, myeloid leukemia, and other/unspecified leukemias

Study	Occupational group	All leukemias		Myeloid leukemia (including AML, CML, unless specified)		Other/unspecified leukemias	
Occupational cohort studies		Overall (No. cases), RR (95 % CI)	Highest exposed (No. cases), RR (95 % CI)	Overall (No. cases), RR (95 % CI)	Highest exposed (No. cases), RR (95 % CI)	Overall (No. cases), RR (95 % CI)	Highest exposed (No. cases), RR (95 % CI)
Wong [20]	Formaldehyde-producing chemical plant	(2), **1.18 (0.13–4.26)	(2), **1.35 (0.15–4.87)^a^				
Levine [57]	Undertakers	4 Observed, 2.5 Expected					
Logue [88]	Radiologists and pathologists	Pathologists: 1.06** Radiologists: 1.55**					
Stroup [86]	Anatomists	(10), **1.5 (0.7–2.7)					
Robinson [61]	Plywood mill workers	1 Observed, 1.7 Expected					
Matanoski [60]	Pathologists	(31), **1.35 (0.92–1.92)					
Ott [66]	3 chemical manufacturing facilities	(2.6), **2 Non-lymphocytic leukemia					
Hall [58]	Pathologists	(3), **1.25 (0.26–3.65)					
Partanen [62]	Wood industry production workers	(2), **1.40 (0.25–7.91)					
Andjelkovich [89]	Automotive iron foundry	(2), **0.43 (0.05–1.57)					
Dell and Teta [67]	Plastics manufacturing, research, development facility	(12), **0.98 (0.50–1.70)	(11), **1.98 (0.99–3.54)^b^				
Band [99]	Pulp and paper workers	(35), **0.85 (0.63–1.13)					
Rapiti [90]	Chemical plant	(1), **1.14 (0.40–7.15) Note: “organic substances” not specific to formaldehyde Note: 90 % CI					
Coggon [48]	6 Formaldehyde-producing or formaldehyde-using factories	(31), **0.91 (0.62–1.29)	(8), **0.71 (0.31–1.39)^c^				
Pinkerton [53]	3 Garment mfg. plants	(24), **1.09 (0.70–1.63)	(12), **1.53^d^	(15), **1.44 (0.80–2.37)	(8), **2.19^d^		
				AML (9), **1.34 (0.61–2.54)	AML (5), **2.02^d^		
Ambroise [91]	Pest-control workers	(1), **4.42 (0.11–24.64)					
Beane Freeman [39]	10 Formaldehyde-producing or formaldehyde-using factories	(116), **1.02 (0.85–1.22)	(29), 1.11 (0.7–1.74)^e^	(44), **0.9 (0.67–1.21)	(10), 1.02 (0.48–2.16)^e^		(9), 1.44 (0.61–3.36)^e^
Linos [92]	Farm-related occupations	(32), 0.70 (0.30–1.20)		AML, CML NS			
	Health-related occupations	0.94, (0.4–2.10)		AML, CML NS			
Hansen [71]	N/A	(23), **1.0 (0.6–1.4)					
Stellman [63]	Woodworkers, wood dust-exposed men	(12), 0.96 (0.54–1.71)^h^					
Blair [70]	N/A		(3), **0.7 (0.2–2.6)^f^		AML (0)^f^		
					CML (1), **0.6 (0.1–5.3)^f^		
Hauptmann [54]	Embalmers and funeral directors	(44), ** 3.0 (1.0–9.5) Non-lymphoid-origin LHPM	(22), **4.0 (1.2–13.2) Non-lymphoid-origin LHPM^g^	(33), **11.2 (1.3–95.6)	(14), **13.2 (1.5–115.4)^g^		

** Note: not all are RR

^a^Hired prior to 1960

^b^Salaried employees (note: this is the only other measure given for LHP malignancies)

^c^“High” exposure, >2.0 ppm

^d^Duration of exposure ≥10 years

^e^Cumulative exposure ≥5.5 ppm-years

^f^“High” exposure

^g^Cumulative exposure >9,253 ppm-h

^h^Formaldehyde exposure only

Leukemia mortality was not elevated overall (SMR 0.91, 95 % CI 0.62–1.29) or in the most highly exposed (i.e., jobs with >2 ppm formaldehyde) segment (SMR 0.71, 95 % CI 0.31–1.39) of the UK formaldehyde producers study [48]. No separate results for myeloid leukemias were presented.

Among other occupational studies, the nested case–control analysis of US embalmers reported odds ratios for myeloid leukemias and for acute myeloid leukemias in the range of 2.0–3.2 for number of embalmings, and for cumulative and peak formaldehyde exposure categories, relative to the referent group that performed <500 career embalmings. However, the underlying sample of death certificates evaluated in this analysis demonstrated no excess of myeloid leukemias: the 29 myeloid leukemias reported in this study generated a proportionate mortality ratio (PMR) of 1.08 (95 % CI 0.70–1.56), and the subset of 20 acute myeloid leukemias generated a PMR of 1.16 (0.71–1.79) [68]. Moreover, there was little evidence of increasing exposure–response trends in the non-reference exposure categories [54]. In the study of US garment workers, the SMR for leukemia deaths was 1.09 (95 % CI 0.7–1.62), based on 24 total leukemia deaths. For the 15 observed myeloid leukemias, the SMR was 1.44 (95 % CI 0.8–2.37), and for the nine acute myeloid leukemias, the SMR was 1.34 (95 % CI 0.66–2.54). In the US garment workers study, SMRs were increased among workers with ≥10 years exposure (SMR for myeloid leukemia 2.19, 95 % CI 0.95, 4.32) [69]^4^ and ≥20 years since first exposure (SMR 1.91, 95 % CI 1.02, 3.27)^5^ [53].

No excesses were observed for all leukemia or for leukemia subtypes among persons classified as exposed to formaldehyde in the population-based case–control studies [70, 71]. In the remaining occupational studies, risk estimates for leukemia compared with the national or regional populations were consistently close to the null value and unstable due to small numbers.

The RR estimate was 5.79 (95 % CI 1.44, 23.25) for leukemia among the combined exposure group of “formaldehyde-exposed and wood-related occupations” in the American Cancer Society Cancer Prevention Study II; however, this result was based on only two deaths. The RR for those with formaldehyde exposure only was 0.96 (95 % CI 0.54–1.71), based on 12 leukemia deaths [63].

### Summary of lymphoma findings

The lymphoma results, including those for chronic lymphocytic leukemia (CLL) when reported separately, are summarized in Table 3. With the exception of Hodgkin lymphoma (HL), there were no overall excesses of the lymphomas among exposed workers in the NCI producers cohort; HL risk was associated with peak exposure, with relative risk reaching 3.96 (95 % CI 1.31–12.02) only at the highest exposure category (≥4.0 ppm), based on 11 deaths. A similar, but weaker, trend was observed for HL and average exposure (RR 2.48, 95 % CI 0.84–2.32) at the highest category [39]. The only overall excess for any of the lymphomas reported in the UK producers study was a weak association for multiple myeloma (MM) in the subgroup classified as mostly highly exposed workers (SMR 1.18, 95 % CI 0.48–2.44) [48]. Quantitative exposure–response findings were not presented.

**Table 3:** Studies of formaldehyde exposure and chronic lymphocytic leukemia, Hodgkin’s lymphoma, non-Hodgkin’s lymphoma, multiple myeloma, and all lymphomas

Study	Occupational group	CLL		HL		NHL		MM		All lymphomas	
Occupational cohort studies		Overall (No. cases), RR (95 % CI)	Highest exposed (No. cases), RR (95 % CI)	Overall (No. cases), RR (95 % CI)	Highest exposed (No. cases), RR (95 % CI)	Overall (No. cases), RR (95 % CI)	Highest exposed (No. cases), RR (95 % CI)	Overall (No. cases), RR (95 % CI)	Highest exposed (No. cases), RR (95 % CI)	Overall (No. cases), RR (95 % CI)	Highest exposed (No. cases), RR (95 % CI)
Wong [20]	Formaldehyde-producing chemical plant			(2), **2.40 (0.27–8.66)	(2), **2.94 (0.33–10.63)^a^						
Stroup [86]	Anatomists			(0), **0.0 (0.0– 2.0)		(2), **0.7 (0.1– 2.5) Note: In study as lymphosarcoma/reticulosarcoma		(6), **2.0 (0.7– 4.4) Note: Includes “Other neoplasms of lymphoid tissue,” “Polycythemia vere,” and “Myelofibrosis”			
Robinson [61]	Plywood mill workers			(2), **3.33 (0.59–10.49) Note: 90 % CI		(3), **2.50 (0.68–6.46) Note: In study as lymphosarcoma/reticulosarcoma 90 % CI		1 observed, 0.6 expected** Note: Includes “Other forms of lymphoma (reticulosis),” “Leukemia and aleukemia,” and “Mycosis fungoides”			
Matanoski [60]	Pathologists			(2), **0.36 (0.04–1.31)		(11), **1.31 (0.66–2.35) Note: In study as lymphosarcoma/reticulosarcoma					
Ott [66]	3 Chemical manufacturing facilities					(2), **2.0		(1), **1.0		(1), **2.6 Lymphocytic leukemia	
Hall [58]	Pathologists			(1), **1.31 (0.03–7.33)							
Partanen [62]	Wood industry production workers			1 Observed		(4), **4.24 (0.68–26.6)				(5), **4.02 (0.87–18.6)	
Dell and Teta [67]	Plastics manufacturing, research, development facility					(3), **0.55 (0.11–1.60) Note: In study as lymphosarcoma/reticulosarcoma	(3), **1.26 (0.26–2.67) Note: In study as lymphosarcoma/reticulosarcoma^b^				
Band [99]	Pulp and paper workers			(7), 0.71 (0.33–1.34)	(4), 1.62 (0.55–3.71)^c^			(12), 0.80 (0.48–1.29)			
Coggon [48]	6 Formaldehyde-producing or formaldehyde-using factories			(6), **0.70 (0.26–1.53)	(1), **0.36 (0.01–2.01)^d^	(31), **0.98 (0.67–1.39)	(9), **0.89 (0.41–1.70)^d^	(15), **0.86 (0.48–1.41)	(7), **1.18 (0.48–2.44)^d^		
Pinkerton [53]	3 Garment mfg. plants	(3), **0.60 (0.12–1.75)		(2), **0.55 (0.07–1.98)		(5), **0.85 (0.28–1.99) Note: In study as lymphosarcoma/reticulosarcoma		(28), **0.97 (0.64–1.40) Note: Includes “Other malignant neoplasms of lymphoid and histocytic tissue”			
Beane Freeman [39]	10 Formaldehyde-producing or formaldehyde-using factories	(36), **1.15 (0.83–1.59)	(10), 1.02 (0.47–2.21)^e^	(25), **1.42 (0.96–2.10)	(4), 1.30 (0.40–4.19)^e^	(94), **0.85 (0.70–1.05)	(21), 0.91 (0.54–1.52)^e^	(48), **0.94 (0.71–1.25)	(15), 1.28 (0.67–2.44)^e^		(50), 1.06 (0.75–1.49)

Study	Occupational group	CLL		HL		NHL		MM		All lymphomas	
Population-based studies		Overall (No. cases), RR (95 % CI)	Highest exposed (No. cases), RR (95 % CI)	Overall (No. cases), RR (95 % CI)	Highest exposed (No. cases), RR (95 % CI)	Overall (No. cases), RR (95 % CI)	Highest exposed (No. cases), RR (95 % CI)	Overall (No. cases), RR (95 % CI)	Highest exposed (No. cases), RR (95 % CI)	Overall (No. cases), RR (95 % CI)	Highest exposed (No. cases), RR (95 % CI)
Linos [92]	Farm-related occupations	NS									
	Health-related occupations	NS									
Boffetta [93]	N/A							(4), 1.8 (0.6–5.7)			
Gerin [94]	N/A			(8), 0.5 (0.2–1.4)		(47), 0.9 (0.5–1.4)	(5), 0.5 (0.1–1.7)^f^				
Heineman [95]	N/A							(144), 1.0 (0.8–1.30)^a^	(41), 1.1 (0.7–1.6)^g^		
Pottern [96]	N/A							(56), 1.1 (0.8–1.6)^a^	(4), 1.6 (0.4–5.3)^g^		
Blair [97]	N/A					(84), 1.2 (0.9–1.7)	(6), 1.3 (0.5–3.8)^h^				
Hansen [71]	N/A			(12), 1.0 (0.5–1.7)^i^		(32), 0.9 (0.6–1.2)^i^					
Tatham [100]	N/A					(93), 1.20 (0.86–1.50)					
Stellman [63]	Woodworkers wood dust-exposed men					(11), 0.92 (0.50–1.68)^j^		(4), 0.74 (0.27–2.02)^j^			
Blair [70]	N/A		(1), 0.6 (0.1–5.3)^k^								
Hauptmann [54]										(81), **1.1 (0.5–2.1) Lymphoid-origin LHPM	(25), **1.0 (0.4– 2.0) Lymphoid-origin LHPM^l^
Tranah [102]	N/A	(90), 1.0 (0.73–1.5)	(29), 1.2 (0.73–1.9)^m^			(757), 1.0 (0.87–1.2)	(205), 0.93 (0.76–1.1)^m^				
Wang [72]	N/A				(24), 1.6 (0.9–3.1)^n^	(203), 1.3 (1.0–1.7)	(8), 1.2 (0.5–2.6)^o^				
Wong [103]	Organic chemicals, chemical fibers and other products	(0)				(12), 0.68 (0.35–1.32)					
	Chemical workers	(0)				(8), 1.60 (0.63–4.05)					
	Wood and furniture workers	(3), 1.50 (0.34–6.70)				(23), 1.54 (0.87–2.70)					

**Note: not all are RR

^a^“Possible exposure” to formaldehyde

^b^Salaried employees (Note: this is the only other measure given for LHP malignancies)

^c^Work duration ≥15 years time since first employed ≥15 years

^d^“High” exposure, >2.0 ppm

^e^Cumulative exposure ≥5.5 ppm-years

^f^Long duration, high concentration exposure level

^g^“Probable exposure” to formaldehyde

^h^“Higher intensity” exposures

^i^Longest work experience had been in companies where there was exposure to formaldehyde at least 10 years before diagnosis

^j^Formaldehyde exposure only

^k^“High” exposure

^l^Cumulative exposure >9,253 ppm-h

^m^“Medium–high average probability” of exposure

^n^Medium and high intensity, medium and high probability of exposure

^o^“Medium–high average” exposure intensity

Results of the nested case–control study of embalmers presented for all neoplasms of lymphoid origin, rather than for non-Hodgkin lymphoma (NHL) or MM specifically, did not suggest an association with any indices of formaldehyde exposure [54]. SMRs for lymphoma were less than 1.0 in the US garment workers study [53]. None of the other occupational cohort studies reported a significantly increased risk of NHL, HL, or MM (Table 3). Risk estimates for NHL, HL, and MM in community-based studies also suggested no association, with RR estimates ranging between 0.5 and 1.3, although positive results were reported in one NHL study from Connecticut [72]. Several community-based studies provided results for NHL subtypes, but there were no consistent associations [59–62].

## Discussion

The main considerations pertinent to assessing epidemiological evidence for a causal relation between formaldehyde exposure and the leukemias or other specific LHM are consistency of findings across studies, evidence for exposure–response associations, accuracy of exposure and health outcome assessment, and minimal confounding and bias. The extent to which exposure assessment in a given study is valid, accurate, and, ideally, permits quantitative dose–response estimation is a critical aspect of research quality. Secondarily, epidemiologic findings suggestive of an association should be interpreted in relation to available evidence of mechanisms of pathogenesis.

The epidemiologic literature provides little or no evidence indicating excess risks overall or exposure–response associations between formaldehyde and any of the LHM, including leukemias, myeloid leukemias, and acute myeloid leukemias. In the majority of occupational cohort studies, which we regard as most informative, specific LHM risk estimates were consistent with the null value, with few exceptions, where the excesses were generally small (i.e., RR < 1.5) and statistically imprecise.

The NCI producers cohort [39] and the nested case–control analysis of the embalmers and funeral directors group [54] found elevated risk estimates based on some exposure metrics compared with an internal reference group. However, the increased relative risk for myeloid leukemia noted in an earlier follow-up of the NCI producers cohort [47] had diminished in the most recent update [39].

The strongest associations for myeloid leukemia observed in this cohort were with peak exposures; whereas cumulative exposure and average exposure intensity were unrelated to risk. As described in the original publication on the exposure assessment of the NCI producers study [73], there was no uniform definition of peak exposure. Instead, peak was defined on a job-specific basis as an excursion (usually of short duration, e.g., <15 min) relative to the estimated average exposure for the job. Moreover, epidemiologic associations of a specific disease with peak exposure can be difficult to interpret in the absence of prior mechanistic support, such as the requirement for acute above-threshold exposures. In general, established human carcinogens show strong and consistent associations between unbiased measures of cumulative exposure and cancer risk, and cumulative exposure is the default dose metric that is mostly used to assess cancer risk for etiologic exposures. A re-analysis of the data from the previous follow-up [47] corroborated the absence of associations with cumulative exposure but also indicated no consistent associations between myeloid leukemia and either duration of time worked at the highest peak or time since highest peak exposure [45]. Findings from similar re-analyses have not been reported for the most recent follow-up. In the other relatively strong occupational cohort study [48], there was no association between formaldehyde exposure and leukemia.

Among the other occupational studies, the US embalmers study generated elevated odds ratios for some formaldehyde exposure metrics [54]. However, as noted by Cole et al. [68], this study has notable limitations—including a lack of overall excess leukemia risks (based on PMR analysis), exposure assessment uncertainties, and a poorly defined study base originating from a convenience sample of death certificates obtained from previous proportionate mortality studies. In the study of US garment workers [53], the only support for an association with formaldehyde was the observation of moderately elevated relative risks for myeloid leukemia associated with long-term exposures and longest follow-up that are very crude exposure metrics correlated with older age. The results of the remaining lower-quality studies are not supportive of an association between formaldehyde exposure and leukemia risk. Another recent review of the literature reached similar conclusions for associations with the leukemias [74].

The pattern of epidemiological results for the lymphomas is inconsistent. In the NCI producers cohort, there were some notably elevated relative risks (in the range of 2.5–4.0) observed for exposure categories of highest peak for HL and MM [39, 47], yet null or at most very small excesses for these diseases were reported in the other studies of occupational formaldehyde exposure.

### Consistency with toxicological and mechanistic evidence

Studies of workers in China have evaluated a potential association between exposure to formaldehyde and a change in one or more blood parameters indicative of hematotoxicity [75–77]. Evidence suggestive of pancytopenia and leukemia-specific chromosome changes was reported from a study of Chinese formaldehyde melamine resin–exposed workers [78]. However, the blood cell parameters among exposed workers were largely within the normal range for Chinese populations [79–82], and the chromosome findings were based on the progeny of circulating stem cells from a small numbers of workers (*n* = 10–12) after 14 days of culture. Overall, the available data do not provide evidence of a clinically or biologically relevant impact on blood cell parameters in humans following exposure to formaldehyde.

Although mechanisms for the development of leukemia or lymphoma following exposure to formaldehyde have been hypothesized [75], they remain speculative. Notably, proposed mechanisms rely heavily on the assumption that formaldehyde can have direct effects on cells or tissues beyond the portal of entry. One fundamental mechanistic question critical to these hypotheses is whether exogenously derived formaldehyde can enter the circulating bloodstream and subsequently damage circulating precursor cells or the bone marrow. Recent experimental research, using extremely sensitive assays with the power to detect as little as one exogenous DNA adduct in 10 billion deoxyguanosines, demonstrated identical endogenously formed DNA formaldehyde adducts in all rat and nonhuman primate non-portal-of-entry tissues, including bone marrow. No exogenous adducts were detected in any distant tissue [83–85]. These considerations call into question the plausibility of causal links between formaldehyde and the LHM.

## Conclusions and recommendations

Existing epidemiologic evidence does not provide convincing support that formaldehyde causes any of the LHMs, including myeloid leukemia. Findings among the highest quality occupational cohort studies are largely null, the positive findings are inconsistent in terms of strength and specificity of association, and there are only isolated instances of exposure–response relations. Epidemiologic evidence from other formaldehyde-exposed occupational cohorts is similarly inconsistent, is often based on small numbers of events, and suffers from a greater likelihood of exposure misclassification and other potential limitations than the two large industrial cohort studies that we regard as highest quality. Available community-based studies, which generally have superior diagnostic classification but poorer quality exposure assessment than in the occupational cohort mortality studies, provide no support for etiologic associations of formaldehyde with any of the LHM.

Although we conclude that a causal connection between formaldehyde exposure and LHM is not supported by existing epidemiologic findings and that the evidence is further weakened by the absence of established carcinogenic mechanisms for the LHM, we nevertheless encourage further epidemiologic research on this topic. We make this recommendation with the caveat that, in order to be informative, further research should offer substantive improvements over the existing body of studies, especially in terms of application of modern diagnostic criteria for specific LHM and individual level quantitative exposure assessment. Well-defined occupational cohort studies should offer the best opportunities to evaluate associations between formaldehyde exposure and LHM risks. Because formaldehyde exposure is ubiquitous, accurately characterizing exposures from the many possible sources, including combustion, household furnishings, automobiles, and consumer products, is essentially impossible. Workplace exposures, on the other hand, are typically substantially higher than exposures from other environmental sources. Continued follow-up of the established high-quality occupational cohorts would be worthwhile, although the scientific yield may be limited because exposure and health outcome misclassification limitations can probably not be remedied. Re-analyses, including sensitivity analysis, of existing datasets may add insight into reported findings, as evidenced by previous re-analyses of the NCI producers cohort data [45]. Specifically, additional statistical analyses of risks of specific LHM in relation to the various exposure metrics in the original NCI producers study [73] are warranted.

A more attractive—but also more complicated and expensive—option would be to enumerate and follow new occupational cohorts exposed to formaldehyde. Professional groups, such as anatomists, pathologists, funeral directors, and embalmers, may be the most appropriate study populations because their exposures are frequent, generally remain at relatively high intensity, and may not be confounded by other potential exposures to leukemogens, such as benzene. Another advantage to studying such professions is that they are comprised of persons with comparable socioeconomic status, a characteristic often associated with baseline rates of LHM in the population.

In contrast, new cohort studies of industrial workers would likely encounter problems related to vastly reduced exposures in large workplaces during the past several decades in many high-income countries, and the resulting reduced capacity to test exposure-related associations rigorously. New occupational cohort studies in developing economies may offer opportunities for further research. Any new occupationally based studies should strive to obtain incidence data with modern LHM classification, and to incorporate valid, thorough exposure assessments for formaldehyde and potential confounders. Cross-sectional and, preferably, prospective investigations of biomarkers of bone marrow toxicity relevant to carcinogenesis that have adequate statistical power would also be worthwhile and might be incorporated into cohort studies where feasible (e.g., on subsets of workers).

In summary, we find insufficient epidemiologic evidence to support a causal relation between formaldehyde exposure and leukemia, including myeloid leukemia. We find no clear evidence of an excess risk of leukemia or myeloid leukemia in any large, well-conducted study. Furthermore, we find the occasional positive associations between various exposure metrics and leukemia or myeloid leukemia risk to be inconsistent, and in some instances, contradictory to results based on more conventional exposure characterization approaches. We also find no epidemiologic basis on which to conclude that formaldehyde causes any of the lymphomas. Further weakening arguments for causal associations is the absence of well-defined plausible models of pathogenesis. Nevertheless, in view of the ubiquitous presence of formaldehyde in the population and experimental evidence indicating high-dose carcinogenic potential, at least for portal-of-entry sites, we recommend improved epidemiologic research on potential risks for the LHM.

## Footnotes

1 Previous publications of the NCI producers study include [40–47].

2 Previous publications of the UK producers study include [49, 50].

3 Previous publications of the US garment workers include [51, 52].

4 95 % CI calculated from data presented in manuscript based on method described by Rothman and Boice [69].

5 95 % CI calculated from data presented in manuscript based on method described by Rothman and Boice [69].
